# Knowledge, Attitudes, and Acceptability of Pre-Exposure Prophylaxis among Individuals Living with HIV in an Urban HIV Clinic

**DOI:** 10.1371/journal.pone.0145670

**Published:** 2016-02-10

**Authors:** Jenani Sarah Jayakumaran, Erika Aaron, Ed J. Gracely, Emily Schriver, Zsofia Szep

**Affiliations:** 1 Drexel University College of Medicine, Philadelphia, PA, United States of America; 2 Department of Infectious Diseases and HIV Medicine, Drexel University College of Medicine, Philadelphia, PA, United States of America; 3 Department of Epidemiology and Biostatistics, Drexel University School of Public Health, Philadelphia, PA, United States of America; David Geffen School of Medicine at UCLA, UNITED STATES

## Abstract

**Introduction:**

Pre-exposure prophylaxis (PrEP) is an effective tool to reduce HIV transmission. The primary objective of this study was to assess awareness of PrEP by individuals living with HIV (HIV+) and acceptance of its use for their HIV negative (HIV-) partners.

**Methods:**

A cross sectional survey was conducted among individuals living with HIV who received care at an urban HIV clinic between January 2013 and June 2013. The survey examined knowledge, attitudes, and acceptability of PrEP, and perception of transmission risk of HIV. Chi-Square test and Fisher's Exact test were used to compare proportions.

**Results:**

Among 206 subjects living with HIV, 15.3% (32) had heard of PrEP. Men who have sex with men (MSM) were more likely to be aware of PrEP than all others (p = 0.003). Once educated about PrEP those who believed PrEP would reduce their partner’s risk for HIV were more likely to recommend PrEP to their partner (p<0.001). 92% of all respondents said they would be “extremely likely/likely” to discuss PrEP use with their provider. Of 159 subjects whose main partner was HIV-, MSM (p = 0.007), male participants (p = 0.044), and those who were consistently taking meds (p = 0.049) were more likely to be aware of PrEP. Those who perceived they were at risk of transmitting HIV (p<0.001) and those who were consistently taking meds (0.049) were more likely to agree that PrEP could reduce the risk of HIV to their partners.

**Conclusion:**

This study illustrates a low awareness of PrEP but once educated the willingness of a cohort of individuals living with HIV to recommend PrEP to their partners. Our findings demonstrate the importance of providers informing their patients living with HIV about PrEP, as these persons are an underutilized link to support the uptake of PrEP by their HIV- partners.

## Introduction

Despite the success of antiretroviral therapy (ART) in dramatically prolonging life expectancy, the incidence of HIV in the United States remains steady with close to 50,000 new infections annually.[1] While behavioral interventions remain an important part of HIV prevention efforts, recently biomedical strategies have moved to the forefront of HIV prevention activities to help reduce new HIV infections. One such biomedical approach is pre-exposure prophylaxis (PrEP) which refers to the use of antiretroviral agents in an HIV-negative individual to reduce the risk of transmission through sexual contact with an individual living with HIV.[2] In July 2012 the FDA approved the daily use of a fixed-dose formulation of tenofovir disoproxil fumarate (tenofovir-DF) and emtricitabine (TDF-FTC) to be used as PrEP for adults at elevated risk of HIV acquisition–a new indication for a medicine already widely used to manage HIV infection.[2] This was coupled with the release of comprehensive clinical practice guidelines for PrEP by the CDC on May 14, 2014. The approval of this drug and subsequent guidelines is a monumental step forward for HIV prevention worldwide.

PrEP has proven to be efficacious in the reduction of risk of HIV transmission in a variety of clinical studies.[3–6] These trials demonstrated the effectiveness of PrEP in the reduction of HIV transmission in men who have sex with men (MSM), women and heterosexual males, and injection drug users (IDU). However, the effıcacy of PrEP has varied widely across trials, and it is affected by adherence to daily doses of TDF–FTC[7]. Implementation studies in France and in England in the MSM population have shown highly successful results with 86% reduction in HIV acquisition [8, 9]. In a recent study from San Francisco Volk et al showed dramatic uptake of PrEP, since its approval in 2012, in a vulnerable MSM population with no new HIV infections[9, 10]. The expansion of PrEP implementation to more diverse settings is needed in order to reduce HIV transmission to vulnerable populations.

While the results of these trials are promising, an improved understanding of the social, cultural, and interpersonal context in relationships which affect attitudes, acceptability and intentions to adopt PrEP among high-risk individuals in the US is needed. Studies that have explored attitudes and acceptance of PrEP in the US have been conducted largely in uninfected MSM [11]. While there have been studies that assess attitudes towards PrEP use in serodiscordant couples [12], or the use of treatment as prevention (TasP) [13], there are no studies to date that explore attitudes and acceptability of the use of PrEP in individuals living with HIV in the US regarding use of PrEP for their partners. Partners are a potential source of support for taking PrEP as has been evidence in serodiscordant couples where relationships can foster treatment adherence and reduce sexual risk behaviors[14]. Additionally, intimacy motivations have been linked to increased uptake of PrEP in order to preserve intimacy during sexual encounters[15]. By involving persons living with HIV as a means of conveying information about PrEP and support for PrEP uptake to their negative partners introduces an opportunity to employ partner-focused HIV prevention interventions.

The primary aim of this study was to examine the knowledge, attitudes, and acceptability of PrEP among individuals living with HIV in an urban HIV clinic in Philadelphia. These results provide an understanding of the perceptions of acceptability to adopt PrEP among persons living with HIV for their partners, which may help inform PrEP implementation programs targeted to this population.

## Methods

A cross sectional survey was conducted between January 2013 and June 2013 among individuals living with HIV who receive medical care at an HIV clinic in Philadelphia, Pennsylvania. The Partnership Comprehensive Care Clinic (The Partnership), Drexel University College of Medicine, provides compressive HIV care to all individuals regardless of their ability to pay.

The IRB of Drexel University College of Medicine approved the protocol for this study. Trained research assistants were responsible for obtaining informed consent, administering the survey orally and recording all responses. Eligible participants were men and women 18 years or older, living with HIV and current patients at The Partnership. Newly diagnosed patients were excluded from the study. Research assistants were responsible for recruiting patients from the waiting room. Eligibility was determined using a brief screening interview where HIV status was self-reported. Participants received a $10 gift card to the hospital cafeteria as compensation for participation in the study. Participants provided written consent using documentation approved by the IRB.

A survey adapted from a validated CDC risk assessment survey [16] consisted of 37 items divided into two sections. The first section collected demographic information as well as risk behavior assessment and perception of risk of HIV transmission to a negative partner; the second examined knowledge and acceptability of PrEP. A scripted definition of PrEP and its effectiveness was provided orally prior to conducting the second section of the survey. The information provided was based on trial results and a comprehensive literature review.[3, 4, 17, 18] Questions examined the willingness of participants to recommend PrEP to their HIV negative partners. Attitudes and potential barriers of recommending PrEP to their negative partners were examined including fear of transmitting HIV, cost, potential side effects, associated condom use, and consistent daily use. Sexual identity was characterized as heterosexual, gay, bisexual, or don’t know. We defined MSM as those gay and bisexual men who identified their partner as male. We employed verbal labels to improve data quality and 5 point Likert scale items for risk perception and acceptability questions. In most cases these were collapsed such that the two highest responses (such as extremely likely and likely), or the two lowest, were the focus on the analysis [19].

Alpha 0.05 was used for all analyses. Using a two-tailed test, 200 subjects would yield 80% power to detect differences on the order of 70% vs. 51% in affirmative responses to an item. Chi-Square test and Fisher's Exact test were used to compare proportions. Data was entered into REDCap (version 6.03); analyses were performed using SAS version 9.3.

## Results

A total of 206 individuals living with HIV were recruited. The median age was 46 years and the mean age of our participants was 44.5 years. Of the participants recruited, 69.9% were heterosexual, 57% were male and 77% were African American. The majority of participants were of lower SES (80% unemployed) with 38% not having graduated from High School or without a GED. [**Table 1]** Of the 118 male participants, 44.9% identified as MSM; of these 90% were African American. Eighty four percent of participants responded that they were “extremely unlikely/unlikely” to transmit HIV to their negative partner.

**Table 1:** Demographic characteristics of subjects (206).

Median Age	46, IQR 15.75
Mean Age	44.5 ±11.4
**Gender**	
Male	57.6% (118)
Female	42.4% (87)
**Sexual Orientation**	
Heterosexual	69.9% (144)
MSM	25.7% (53)
Lesbian	2.9% (6)
Questioning or Don’t Know	1.5% (3)
**Race**	
Caucasian	9.3% (19)
African American	77.1% (158)
Hispanic or Latino	5.9% (12)
Mixed or other	7.9% (16)
**Highest Level of Education**	
Less than High School	38.4% (79)
High School/GED	31.6% (65)
More than High School	30.1% (62)
**Employment Status**	
Full-time or Part-time	18.9% (39)
Unemployed	80.1% (165)
**Health Insurance Status**	
Insured	90.7% (185)
Uninsured	9.3% (19)
**Missed Medicine in the Past 7 days**	
Yes	32.5% (62)
No	67.5% (129)
**Condom use during last sexual encounter**	
Yes	71.6% (144)
No	28.4% (57)
**Condom use during last month**	
Every time	37.0% (74)
Not every time	63.0% (126)

Only 15.3% (32) had heard of PrEP: MSM were more likely to have heard of PrEP (n = 15, 28.8%) (p = 0.003) and men were more likely than women (p = 0.011). Once a scripted definition of PrEP was provided, 88.8% said they would be “extremely likely/likely” to recommend PrEP to a negative partner. Those who believed PrEP would reduce their partner’s risk for HIV were more likely to recommend PrEP to their partner (p<0.001). [**Table 2**] There were no significant differences in likelihood of recommending PrEP to an HIV negative partner based on race, education, employment, health insurance, age, condom use in the past month, or perceived risk of transmitting HIV.

**Table 2:** Factors Associated with PrEP Awareness and Acceptance (206).

**Awareness: Have you ever heard of pre-exposure prophylaxis (PrEP) before?**				
	** **	**Yes N (%)**	**No N (%)**	**p-value**
**Sexual Orientation(Non MSM includes heterosexual, lesbian, and questioning/don’t’ know)**	Non- MSM (153)	17(11.9)	135 (89%)	**0.003**
	MSM (53)	15 (28.8%)	43(74.1)	
**Gender**	Male (117)	25 (21.4)	92 (78.6)	**0.011**
	Female (86)	7 (8.1)	79 (91.9)	
**Condom use during last sexual encounter**	Yes (144)	19(13.2)	125(86.8)	0.093
	No (57)	13(22.8)	44 (77.2)	
**Have you missed any medicine in past 7 days?**	Yes (62)	51 (82.3)	11 (17.7)	0.062
	No (129)	118(91.5)	11(8.5)	
**Acceptance: How likely would you be to recommend PrEP to an HIV—partner?**				
		**Extremely Likely/Likely**	**Extremely Unlikely/ Unlikely/Unsure**	**p-value**
**Education**	Finished High School (134)	122 (91.0)	12 (9.0)	0.170
	Did not finish High School (72)	61 (84.7)	11 (15.3)	
**I believe PrEP will reduce the risk of HIV transmission to my partner**	Strongly Agree/Agree (170)	160 (94.1)	10 (5.9)	**<0.001**
	Strongly Disagree/ Disagree/ Undecided (35)	23 (65.7)	12 (34.3)	

Boldface indicates statistical significance (p<0.05)

Of the 206 participants, 164 had a main partner who was HIV negative or currently had no main partner. Main partner was defined as the individual whom the identified as his or her primary sexual contact. In this subgroup those who were MSM (p = 0.007), male participants (p = 0.044), and those who were consistently taking prescribed medications (defined as 100% adherence in past 7 days) (p = 0.049) were more likely to have heard of PrEP. Those who agreed PrEP could reduce their partner’s risk of HIV perceived themselves at risk of transmitting HIV (p<0.001) and were consistently taking meds (p = 0.049). **[Table 3]** There were no significant differences in the likelihood of recommending PrEP based on condom use in the past month.

**Table 3:** Factors associated with PrEP Awareness and Acceptance in individuals living with HIV with HIV- Main Partners (164).

**Awareness: Have you ever heard of PrEP before?**				
	** **	**Yes N (%)**	**No N (%)**	**p-value**
**Sexual Orientation(Non MSM includes heterosexual, lesbian, and questioning/don’t’ know)**	Non- MSM (116)	14 (12.1)	102 (87.9)	**0.007 **
	MSM (43)	13 (30.2)	30 (69.8)	
**Gender**	Male (91)	20 (22.0)	71 (78.0)	**0.044**
	Female (70)	7 (10.0)	63 (90.0)	
**Age Group**	<35 (38)	10 (26.3)	28 (73.7)	0.068
	≥35 (124)	17 (13.7)	107 (86.3)	
**Have you missed any medicine in past 7 days?**	Yes (51)	4 (7.8)	47 (92.2)	**0.049**
	No (99)	20 (20.2)	79 (79.8)	
**Condom use during last sexual encounter**	Yes (119)	17 (14.3)	102 (85.7)	0.118
	No (40)	10 (25.0)	30 (75.0)	
**Acceptance: How likely would you be to recommend PrEP to an HIV- partner)?**				
	** **	**Extremely Likely/Likely**	**Extremely Unlikely/ Unlikely/Unsure**	**p-value**
**I believe that PrEP will reduce the risk of HIV transmission to my partner**	Strongly Agree/ Agree (137)	128 (93.4)	9 (6.6)	**<0.001 **
	Strongly Disagree/ Disagree/ Undecided (26)	17 (65.4)	9 (34.6)	
**Have you missed any medicine in past 7 days?**	Yes (53)	43 (81.1)	10(18.9)	**0.049 **
	No (99)	91 (91.9)	8(8.1)	
**Condom use in last month**	Every time (64)	54 (84.4)	10 (15.6)	0.196
	Not every time (100)	91 (91.0)	9 (9.0)	

Boldface indicates statistical significance (p<0.05). Some variables have missing data.

Respondents expressed positive feelings about their partner’s taking PrEP: 82.9% (171) believed that it would reduce the risk of HIV to their partner(s) and 83.7% (172) would have more hope about the future if their partner took PrEP. 95.3% (196) believed PrEP should be available and free, and 92% said they would want to discuss PrEP with their provider. When determining acceptability and barriers, 70% (144) said even if PrEP required a daily pill, this would not prevent them from recommending PrEP to their partner(s). Furthermore, 58% said they would still be “extremely likely/likely” to recommend PrEP to their HIV negative partners if side effects included headache, abdominal pain, and weight loss. An overwhelming 76.8% of participants said they would be “extremely likely/likely” to use condoms every time they had vaginal or anal intercourse if their partner was taking PrEP; 54.1% said they would be “a little less likely/less likely” to worry about transmitting HIV to a negative partner.

## Discussion

We examined the knowledge and acceptability of PrEP among a cohort of individuals living with HIV in an urban clinic, a population who are an essential component to the success of PrEP use by their HIV negative partners. The participants had low knowledge of PrEP with only 15.3% of the entire cohort having had a previous knowledge of PrEP and 29% of MSM had a previous knowledge of PrEP (P<0.003). However, once educated about PrEP effectiveness, the majority (88.8%) said they would recommend PrEP to their partners. Not surprisingly, expressed belief that PrEP would reduce the risk of HIV transmission was a significant predictor of willingness to recommend PrEP to a partner in the overall sample. Furthermore, belief that they were at risk of transmission to an HIV- partner predicted willingness to recommend in the subset who did not have a main partner living with HIV. Perception of risk play an important role in adherence and thus in the effectiveness of PrEP as an prevention tool [8]. Educating persons who are living with HIV about PrEP may be an effective intervention in supporting partner uptake of PrEP and thus potentially decreasing the incidence of HIV. Targeting persons who perceive themselves at risk of transmitting HIV may yield a higher uptake of PrEP for their partners. In a US based study MSM viewed main partners as a potential source of support for taking PrEP[18]. Involving persons living with HIV as a means of conveying information about PrEP to their negative partners introduces an opportunity to employ partner-focused HIV prevention interventions.

Participants in this study who identified themselves as MSM were more likely to have previous knowledge of PrEP, however only 29% were aware of PrEP. This remained true among a subgroup of MSM that identified having an HIV negative main partner. MSM have been identified as a high-risk group for which PrEP may be an appropriate prevention option. MSM represent 65% of incident HIV infections in the US between 2008 and 2011 [20]. Results from other studies have demonstrated low awareness of PrEP among MSM but a relatively high level of willingness to use PrEP [11]. MSM who report high risk sexual behavior are more than twice as likely to report willingness to use PrEP. Those who report low perceptions of risk are more likely to reject the option of PrEP[11]. It is important to understand how risk is perceived. Brooks et al reported on perceptions and intentions to adopt PrEP among Black MSM in Los Angeles. Similar to our findings, a very low percentage of participants were aware of PrEP (33%) and more than half (60%) indicated a high intention to adopt PrEP. These findings concur with ours as 86.8% of our MSM population said they would be “extremely likely/likely” to accept a partner’s use of PrEP. Structural stigma has been shown to negatively impact risk behaviors. Lower structural stigma has been associated with decreased odds of condomless anal intercourse, increased odds of awareness of PrEP, and increased comfort discussing male–male sex with providers.[21].

Women living with HIV in this study were less likely than men to have heard of PrEP (p = 0.013). Research regarding PrEP use specific to women who are HIV negative in the US is limited and needs further exploration. However, it is clear that women are accessing PrEP as data from 55% of US pharmacies found that women represented half (47.7%) of PrEP users.[22] In two studies, ADAPT and TDF2 open-label extension study, which closely replicated a real-world setting rather than a clinical trial, where participants did not receive financial compensation and all were aware they were taking an active drug, adherence was excellent in women [23].However, results from the VOICE study suggest that social barriers such as societal stigma may have led to the demonstrated low levels of adherence in this trial[24]. A better understanding of the social and structural influences on women and the uptake of PrEP is needed. Understanding women’s perceptions of PrEP is helpful when counseling their partners with HIV in prevention methods.

There is a lack of understanding as to the social and structural influences that impact PrEP acceptability. A recent review of research on factors influencing acceptability of PrEP revealed great variability in studies; some studies report an association with lower levels of education and a greater willingness to use PrEP, while other studies have found no association between willingness and income or education[25]. The majority of the participants in our study were unemployed (80%) and had lower levels of education (40% had less than high school education). This is a reflection of the population of persons living with HIV in the US reported from 2006–2007: HIV prevalence was 2.8% among participants with less than a high school education compared with 1.2% among those with more than a high school education, 2.6% among participants who were unemployed compared with 1.0% among those who were employed, and 2.3% among participants with annual household incomes at or below the poverty level compared with 1.0% among those with incomes above the poverty level[26]. It is important to understand the social influences that affect the acceptability of PrEP for persons living with HIV

Most participants in this study were “extremely likely/likely” to discuss PrEP with their medical provider. In order to facilitate PrEP adoption medical providers need to offer accurate information on the benefits of PrEP to their patients[17]. Previous studies have demonstrated limited prescribing intentions of PrEP by providers. Impediments to wider PrEP use could be overcome by enhancing provider education as to the benefits of PrEP and the ease of prescribing this to their patients. [27]; Communication by providers with their patients living with HIV is recommended as a proportion of this patient population is in regular contact with the healthcare system and represents a unique bridge to HIV negative partners. In the US however, only 40% of persons living with HIV are retained in HIV care and only 19% of those in care have an undetectable viral load[28]. This presents a public health challenge to connect with persons with HIV who are out of care for their own health as well as for protection of their partners from HIV transmission

### Limitations

Due to the small number of participants and the methodology employed, the generalizability of our results is limited. The study was performed at an urban HIV clinic with a large portion reporting unemployment and less than high school education, thus limiting the generalizability of our results to other settings. In addition, the inferences on women are outside the scope of this study but are much needed in the literature. A larger study is necessary to assess the acceptance of PrEP among women living with HIV in the US. Our findings relied on self-report data, which could be subject to self-report bias. However, the anonymous nature of the survey, which was conducted by outside research assistants who were not involved in the participants care, may have limited this potential bias. Furthermore, this study does not consider HIV negative casual partners who are a vulnerable and important population to consider.

## Conclusions

The results of this study provide an understanding of the perceptions and intentions of persons living with HIV to recommend PrEP to their partners. This study is novel in its approach in assessing attitudes towards the use of PrEP among persons living with HIV, a population that has not been studied and is an underutilized link to HIV negative partners. While knowledge was limited about the utilization of PrEP, once educated, individuals living with HIV were interested in learning new ways to protect their partners. By involving the individual living with HIV introduces an opportunity to provide support for delivering PrEP in a relationship-focused and partner-inclusive format.

The limited knowledge of PrEP among participants in this study suggests that greater efforts are needed to raise awareness and disseminate information to persons who are living with HIV. A challenge remains for health care providers to be well educated about PrEP and inform their patients living with HIV about its benefits. Although clinical trials have illustrated that PrEP is a highly effective HIV prevention method, the acceptability of PrEP will ultimately determine the success of this prevention tool. Important challenges remain in fully implementing PrEP; limited information exists on the attitudes, and beliefs about PrEP and intentions to adopt PrEP in the US. Structural barriers of PrEP implementation such as cost and insurance coverage is an important next step to informing implementation science initiatives. A better understanding is needed of the social, structural influences, intimacy motivations, and the role of health providers have on the impact of acceptability and uptake of PrEP.

## Supporting Information

S1 Dataset (CSV)
